# A growth factor-free culture system underscores the coordination between Wnt and BMP signaling in Lgr5^+^ intestinal stem cell maintenance

**DOI:** 10.1038/s41421-018-0051-0

**Published:** 2018-09-04

**Authors:** Yehua Li, Yuan Liu, Bofeng Liu, Jilian Wang, Siting Wei, Zhen Qi, Shan Wang, Wei Fu, Ye-Guang Chen

**Affiliations:** 1 The State Key Laboratory of Membrane Biology, Tsinghua-Peking Center for Life Sciences, School of Life Sciences, Tsinghua University, Beijing, 100084 China; 2 Tsinghua-Peking Center for Life Sciences, School of Life Sciences, Tsinghua University, Beijing, 100084 China; 3 Department of General Surgery, Peking University Third Hospital, Beijing, 100191 China

## Abstract

Lgr5^+^ intestinal stem cells (ISCs) drive the fast renewal of intestinal epithelium. Several signaling pathways have been shown to regulate ISC fates. However, it is unclear what are the essential signals to sustain the ISC self-renewal. Here we show that coordination between Wnt and BMP signaling activity is necessary and sufficient to maintain Lgr5^+^ ISCs self-renewal. The key function of R-spondin1 is to achieve a high activity of Wnt signaling in the organoid culture. Using the GSK3 inhibitor CHIR-99021 and the BMP type I receptor inhibitor LDN-193189, we can maintain Lgr5^+^ ISCs without growth factors in vitro. Our results define the basic signaling pathways sustaining Lgr5^+^ ISCs and set up a convenient and economical culture system for their in vitro expansion. This work also set up an example for growth factor-free culture of other adult stem cells.

## Introduction

Lgr5^+^ intestinal stem cells (ISCs) residing at the intestinal crypt bottom are required for the fast renewal of intestinal epithelium. Their fates are determined by environmental cues, including Wnt^1–3^, Notch^4,5^, Hippo^6,7^, bone morphogenetic protein (BMP)^8–10^, and epidermal growth factor (EGF)^11^. The establishment of in vitro culture system for Lgr5^+^ ISCs has greatly facilitated our understanding of how these stem cells are maintained and their fate is determined^12^. Requirement of EGF, Noggin, and R-spondin1 in this culture system emphasizes the importance of those signaling pathways. However, it was reported that EGF is dispensable for Lgr5^+^ ISC maintenance in vitro^13,14^, and the exact function of R-spondin1 in Lgr5^+^ ISC maintenance is unclear^15–17^. By establishing a novel culture system with chemicals, we not only established a convenient and economic method to expand Lgr5^+^ ISCs, but also identified the basic signaling pathways for Lgr5^+^ ISC maintenance.

## Results and discussion

### Wnt activation and BMP inhibition are necessary and sufficient for Lgr5^+^ ISCs maintenance in vitro

**Fig. 1:**
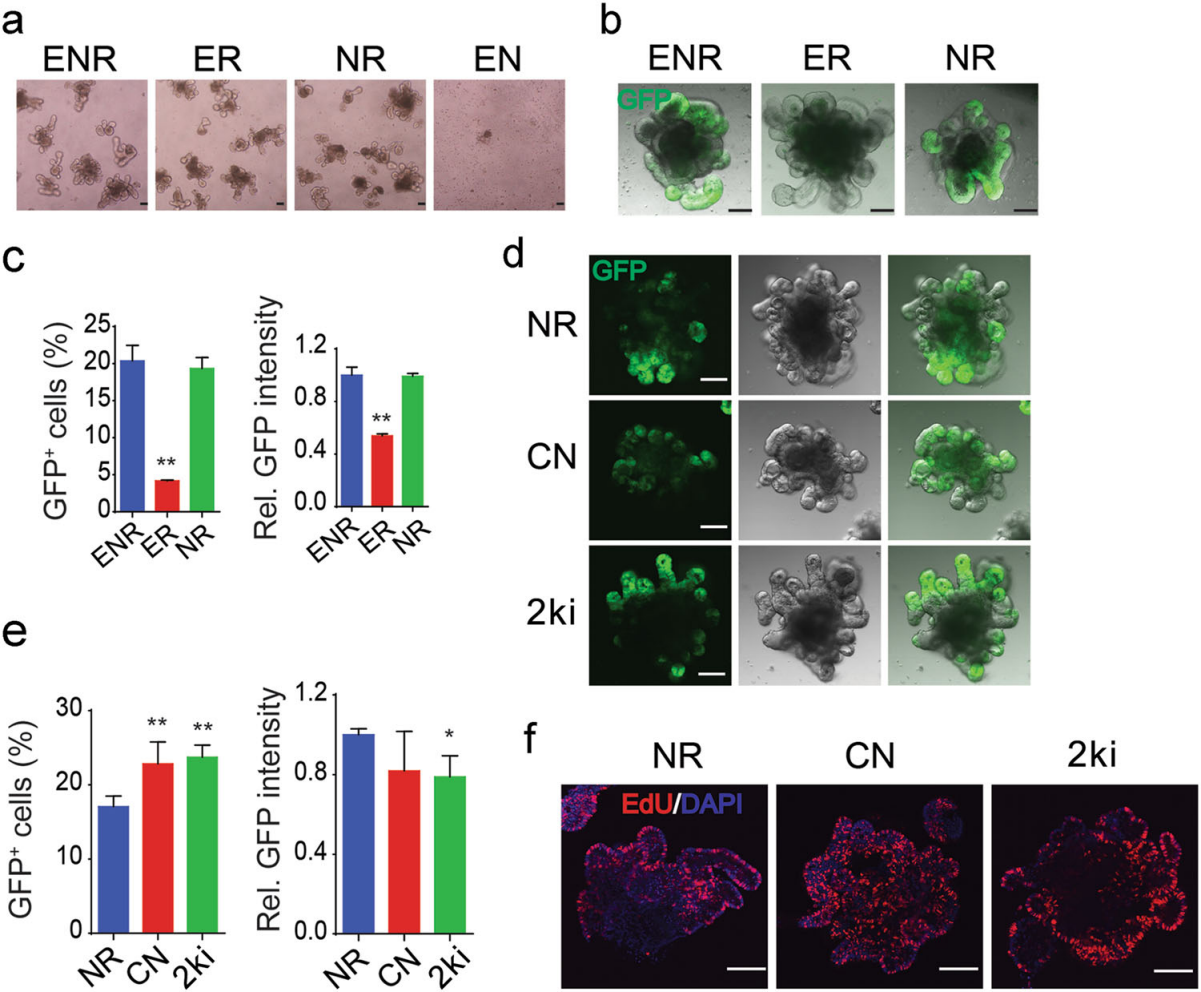
Wnt activation and BMP inhibition are sufficient to maintain Lgr5^+^ ISCs. **a** Representative bright-field images of small intestinal organoids cultured with EGF, Noggin and R-spondin1 (ENR); EGF and R-spondin1 (ER); Noggin and R-spondin1 (NR); and EGF and Noggin (EN) for 7 days. **b**, **c** GFP fluorescence images (**b**) and FACS analysis of GFP expression (**c**) of small intestinal organoids cultured with ENR, ER and NR for 7 days. **d**, **e** GFP fluorescence images (**d**) and FACS analysis of GFP expression (**e**) of small intestinal organoids cultured with NR, CHIR and Noggin (CN) and CHIR and LDN (2ki) for 7 days. **f** EdU staining of proliferating cells in small intestinal organoids cultured with NR, CN and 2ki for 7 days. Scale bars, 100 μm. **P < 0.01 and *P < 0.05 analyzed by two-way ANOVA test. Error bars, s.d. *n* = 3 mice in **c** and *n* = 4 mice in **e**. Refer to Supplementary Figure 1 and 2 for detailed analysis

To identify the minimal essential requirement of external signals for Lgr5^+^ ISCs maintenance, we used various combinations of growth factors to culture small intestinal crypts derived from *Lgr5-EGFP-IRES-creERT2* mice^18^, and found that R-spondin1 could sustain organoid survival for 2 weeks, while EGF and Noggin were dispensable (Fig. 1a, Supplementary Fig. S1a). However, removal of the BMP antagonist Noggin led to a dramatic decrease of Lgr5^+^ ISCs, whereas EGF had no contribution for Lgr5^+^ ISCs maintenance (Fig. 1b, c, Supplementary Fig. S1b, c). These data demonstrated the essential role of R-spondin1 and Noggin, but not EGF, in the in vitro maintenance of Lgr5^+^ ISCs.

R-spondin1 is a Wnt agonist^16,17^, but it cannot be replaced by Wnt ligands in organoid culture. On the contrary, loss-of-function mutations of Adenomatosis Polyposis Coli (APC), the negative regulator of Wnt/β-catenin signaling, could sustain organoid culture without R-spondin1^19,20^. These results prompted us to hypothesize that organoid growth might need a high activity of Wnt/β-catenin signaling, which could not be achieved by Wnt ligand alone. During our study, we found 5 or 10 μM GSK3 inhibitor CHIR-99021 (CHIR) treatment could activate Wnt/β-catenin signaling to the similar level of Wnt plus R-spondin1 with Topflash-luciferase reporter assay in HEK293T cells (Supplementary Fig. S1d). To test our hypothesis, we used different doses of CHIR to culture organoids and found that low dose CHIR could not sustain organoids survival (Supplementary Fig. S1e). Consistently, F106A mutation of R-spondin1, which lost its Wnt activation ability^21^ (Supplementary Fig. S1d), could not support organoid culture. When CHIR concentration was increased to 10 μM, it alone could maintain the organoid survival and proliferation (Supplementary Fig. S1e, f), although GFP^+^ ISCs were very rare in the organoids (Supplementary Fig. S1g, h). Although both 5 and 10 μM CHIR could activate Wnt signaling to the similar level in the reporter assay (Supplementary Fig. S1d), only 10 μM CHIR could maintain organoid survival (Supplementary Fig. S1e). In line with it, only 10 μM CHIR could enhance the Wnt target gene expression to the similar level of R-spondin1 treatment, and the Lgr5^+^ ISC signature gene expression was significantly higher than treated with 5 μM CHIR in cultured organoids (Supplementary Fig. S1i). These results suggest that the major function of R-spondin1 in organoid culture is to maintain a high activity of Wnt/β-catenin signaling.

### Lgr5^+^ ISCs can be maintained in a growth factor-free culture system

As 10 μM CHIR could substitute R-spondin1 to sustain organoids survival, we tested if CHIR could replace R-spondin1 and cooperate with Noggin to maintain Lgr5^+^ ISCs. As shown in Fig. 1d–f, CHIR and Noggin were able to sustain Lgr5^+^ ISC self-renewal and proliferation. Then we assessed if Noggin could be replaced by the BMP type I receptor inhibitor LDN-193189 (LDN) to support Lgr5^+^ ISC self-renewal in the presence of CHIR. As shown in Fig. 1d, e, LDN and CHIR were sufficient to sustain Lgr5^+^ ISCs without addition of growth factors, and LDN increased Lgr5^+^ ISCs in a dose dependent manner (Supplementary Fig. S2a).

As 0.5 μM LDN did not further increase the percentage of Lgr5^+^ ISCs, 10 μM CHIR and 0.2 μM LDN were employed for organoid culture from crypts, and we named this culture system as 2ki culture system. The morphology of the most 2ki-cultured organoids was similar to that of ENR-cultured ones, while some were round shaped, alike the ones cultured with ENR plus Wnt3a (Fig. 1d, Supplementary Fig. S2b). In line with that, 10 μM CHIR induced a high Wnt signaling activity (Supplementary Fig. S1d). Further, the colony number was slightly higher in the 2ki culture system (Supplementary Fig. S2b). Fluorescence-activated cell sorting (FACS) analysis showed that 2ki culture yielded more GFP^+^ cells, but GFP intensity was slightly lower (Fig. 1e). EdU labeling indicated that the cell proliferation ability was similar in the two culture systems (Fig. 1f). Immunoblotting analysis revealed that activation of AKT and p38 was attenuated, and the β-catenin level was slightly increased, while the Smad1/5 phosphorylation and YAP levels remained similar in 2ki-cultured organoids compared to ENR-cultured ones (Supplementary Fig. S2c).

We have previously shown that blebbistatin could promote survival of crypts^22^, and Yin et al. demonstrated that valproic acid (VPA) increased Lgr5^+^ ISCs in ENR-cultured organoids^14^. We then examined their effect in the 2ki culture system and found that blebbistatin markedly increased the organoid number (Supplementary Fig. 2d), while VPA had no effect at 0.5 μM, even slightly reduced the number at 1 μM (Supplementary Fig. S2e).

**Fig. 2:**
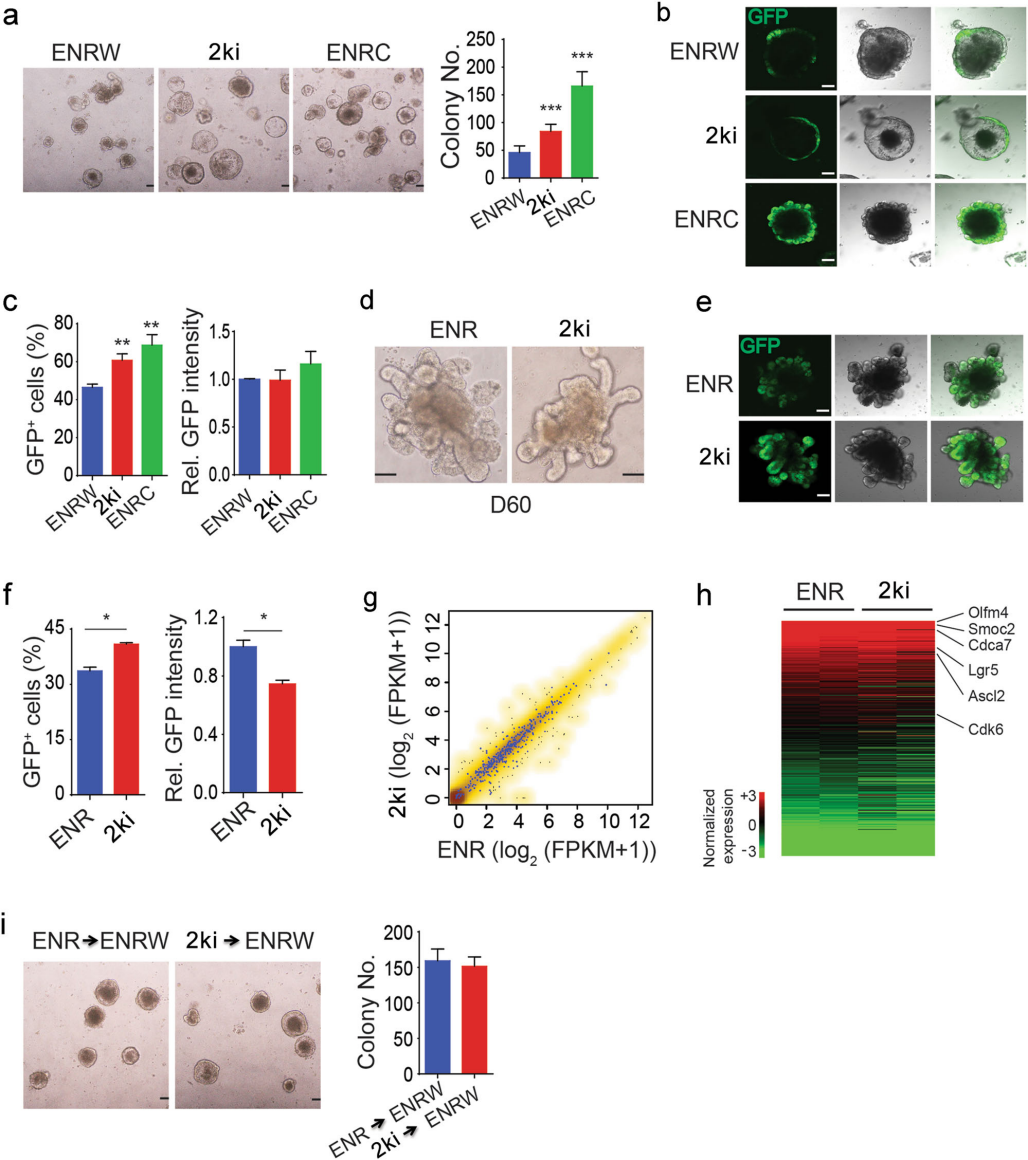
CHIR and LDN together maintain Lgr5^+^ ISCs. **a** Representative images and quantitation of colony numbers of 5,000 FACS-isolated Lgr5-GFP cells cultured with ENR plus Wnt3a (ENRW), CHIR and LDN (2ki) or ENR plus CHIR (ENRC) for 7 days. **b**, **c** GFP fluorescence images (**b**) and FACS analysis of GFP expression (**c**) of organoids derived from single small intestinal Lgr5-GFP cells cultured with ENRW, 2ki and ENRC for 7 days. **d**–**f** Representative bright-field (**d**), GFP fluorescence images (**e**), and FACS analysis of GFP expression (**f**) of small intestinal organoids cultured with ENR and 2ki for 2 months. **g** Scatter plots comparing the gene expression between ENR and 2ki organoids (spearman correlation is 0.976). The Lgr5^+^ ISC signature genes are showed in blue dots. **h** Heat maps showing the RNA expression of Lgr5^+^ ISC signature genes. **i** Lgr5-GFP cells sorted from organoids cultured with ENR or 2ki for 2 months were shifted to ENRW culture for 7 days, and then representative images and quantitation of colony numbers were shown. Scale bars, 100 μm. ***P < 0.001, **P < 0.01, and *P < 0.05 analyzed by two-way ANOVA test. Error bars, s.d. *n* = 4 wells from 2 mice in **c** and **f**, *n* = 9 wells from 3 mice in **a**, and *n* = 6 wells from 2 mice in **i**. Refer to Supplementary Figure 3 for detailed analysis

We further found that the 2ki system could not only culture single Lgr5^+^ cells to form organoids but also increase the number of formed organoids, even more efficient than ENR plus Wnt3a (ENRW) culture (Fig. 2a). In the single cell-derived organoids, more GFP^+^ cells were detected in the 2ki culture than that in the ENRW culture (Fig. 2b, c).

CHIR and LDN were able to maintain organoids and Lgr5^+^ ISCs for a long time, and the morphology was similar in 2ki organoids and ENR ones after 10 passages in 2 months (Fig. 2d). The percentage of Lgr5^+^ ISCs was higher in 2ki organoids, although the GFP intensity was slightly lower (Fig. 2e, f, Supplementary Fig. S2g). Transcriptome analysis also indicated that the gene expression profile, including the Lgr5^+^ ISC signature genes, was similar in the GFP^+^ ISCs cultured with ENR or 2ki (Fig. 2g, h). Furthermore, the two culture systems maintained the Lgr5^+^ ISCs with similar colony formation abilities (Fig. 2i), and the cells cultured with 2ki showed a normal karyotype after 20 passages (2*n* = 40 chromosomes; Supplementary Fig. S2f). These results together indicate that CHIR and LDN can maintain Lgr5^+^ ISCs in a long term with normal characteristics.

### Differentiation toward secretory cells are reserved in 2ki cultured organoids

**Fig. 3:**
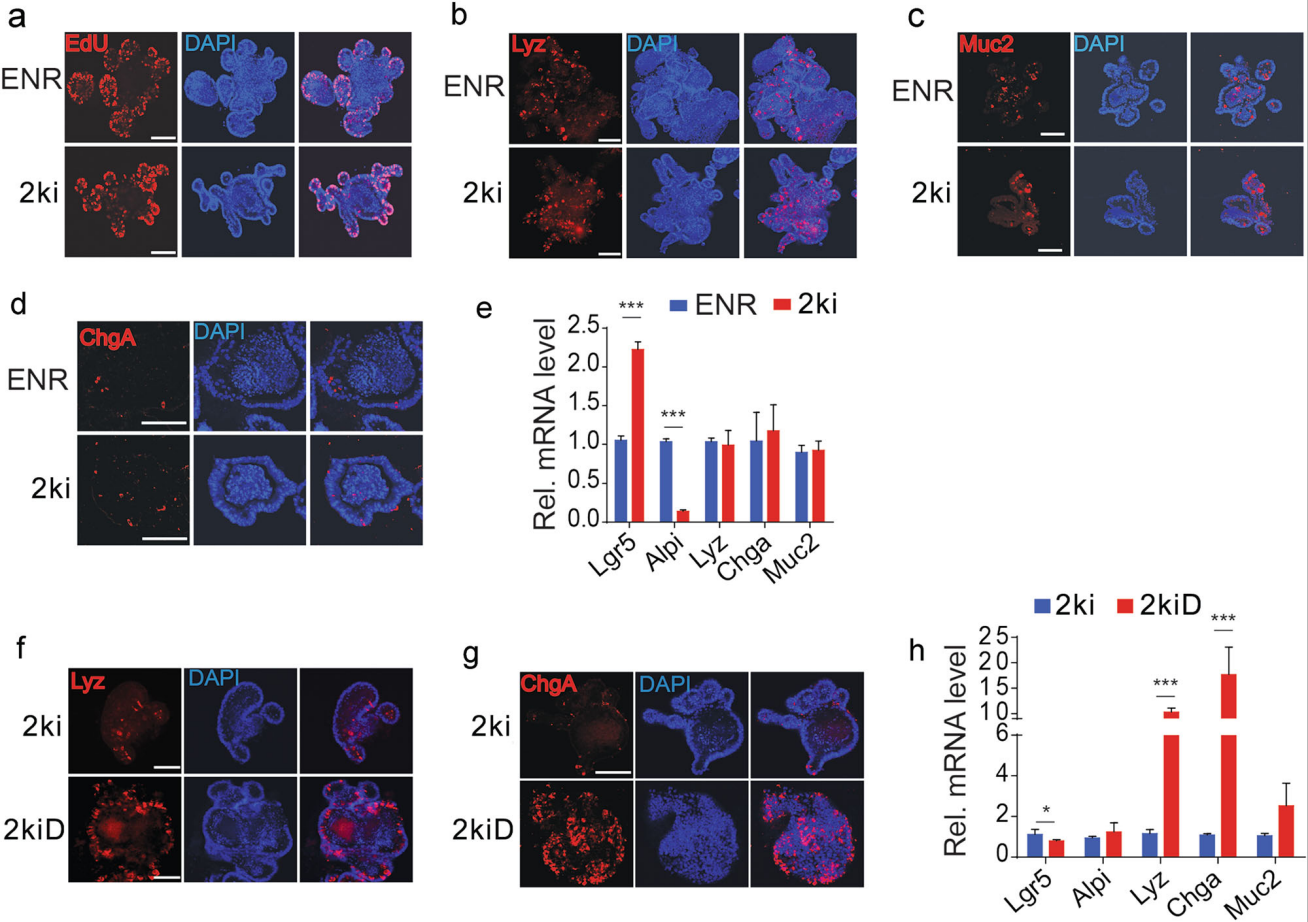
The organoids cultured with either ENR or 2ki have similar secretory differentiation. **a**–**d** EdU staining of proliferation cells (**a**), Lyz staining of Paneth cells (**b**), Muc2 staining of goblet cells (**c**), and ChgA staining of enteroendocrine cells (**d**) in organoids cultured with ENR or 2ki for 2 months. **e** Expression of stem cell and differentiated cell marker genes in organoids cultured with ENR or 2ki. **f**, **g** Lyz staining of Paneth cells (**f**) and ChgA staining of enteroendocrine cells (**g**) in organoids cultured with 2ki or 2ki plus DAPT (2kiD) for 4 days. **h** Gene expression of stem cell and differentiated cell marker genes in organoids cultured with 2ki or 2kiD. Scale bars, 100 μm. ***P < 0.001 and *P < 0.05 analyzed by Student’s *t*-test. Error bars, s.d. (*n* = 3 independent experiments)

ENR organoids contain all intestinal epithelial cell types, including Lgr5^+^ stem cell, proliferating transit amplifying cell (TA cell), enterocytes (Alpi^+^), Paneth cells (Lyz^+^), goblet cells (Muc2^+^), and enteroendocrine cells (ChgA^+^)^12^. EdU staining revealed that cell proliferation was similar in ENR and 2ki organoids (Fig. 3a), indicating that the 2ki culture system can efficiently maintain TA population. Differentiation of the secretory cells (Paneth cells, goblet cells, enteroendocrine cells) was normal in 2ki organoids as indicated by the staining of their markers Lyz, Muc2, and ChgA, respectively (Fig. 3b–d). Quantitative PCR (qPCR) anaylsis of marker gene expression also showed higher expression of Lgr5 and similar levels of Lyz, Chga, and Muc2 in 2ki organoids compared to ENR organoids (Fig. 3e). However, the enterocyte marker Alpi was much lower in 2ki organoids, indicating that the secretory cell differentiation was normal in 2ki culture, while enterocyte differentiation was attenuated. Moreover, consistent with its function in ENR culture^14^, the Notch signaling inhibitor DAPT promoted differentiation of Paneth cells (Lyz^+^) and enteroendocrine cells (ChgA^+^) in 2ki culture (Fig. 3f–h).

A high Wnt activity is needed to culture colon crypts^12^. Then we tested whether CHIR and LDN were sufficient to culture colon crypts. As shown in Supplementary Fig. S3a, 2ki culture markedly increased the colony number when compared to ENRW culture. Cell proliferation, GFP^+^ ISC number and GFP intensity were also dramatically increased in 2ki culture (Supplementary Fig. S3b-d). More Lgr5^+^ ISCs in 2ki culture could be due to higher Wnt signaling activity mediated by CHIR. Indeed, qPCR showed that in addition to higher mRNA levels of the ISC markers *Lgr5* and *Ascl2*, the mRNA levels of the Wnt targets *Axin2* and *MMP7* were higher in 2ki organoids than ENRW organoids (Supplementary Fig. S3e). In accordance with the results obtained with small intestine organoids, the mRNA levels of the secretory cell markers *Chga* and *Muc2* were similar in the two culture systems, while the enterocyte marker *Alpi* was lower in 2ki organoids (Supplementary Fig. S3e). Immunofluorescence analysis also showed that Muc2 protein levels were similar in the two culture systems (Supplementary Fig. S3f). Single-cell colony formation assay of colon Lgr5^+^ ISCs showed that 2ki was much better than ENRW to culture colon ISCs (Supplementary Fig. S4a). Colon Lgr5^+^ ISCs could be maintained in 2ki condition for more than 2 months after 10 passages (Supplementary Fig. S4b-d), and retained the differentiation ability (Supplementary Fig. S4e).

### Wnt and BMP coordinate with each other to maintain Lgr5^+^ ISCs

**Fig. 4:**
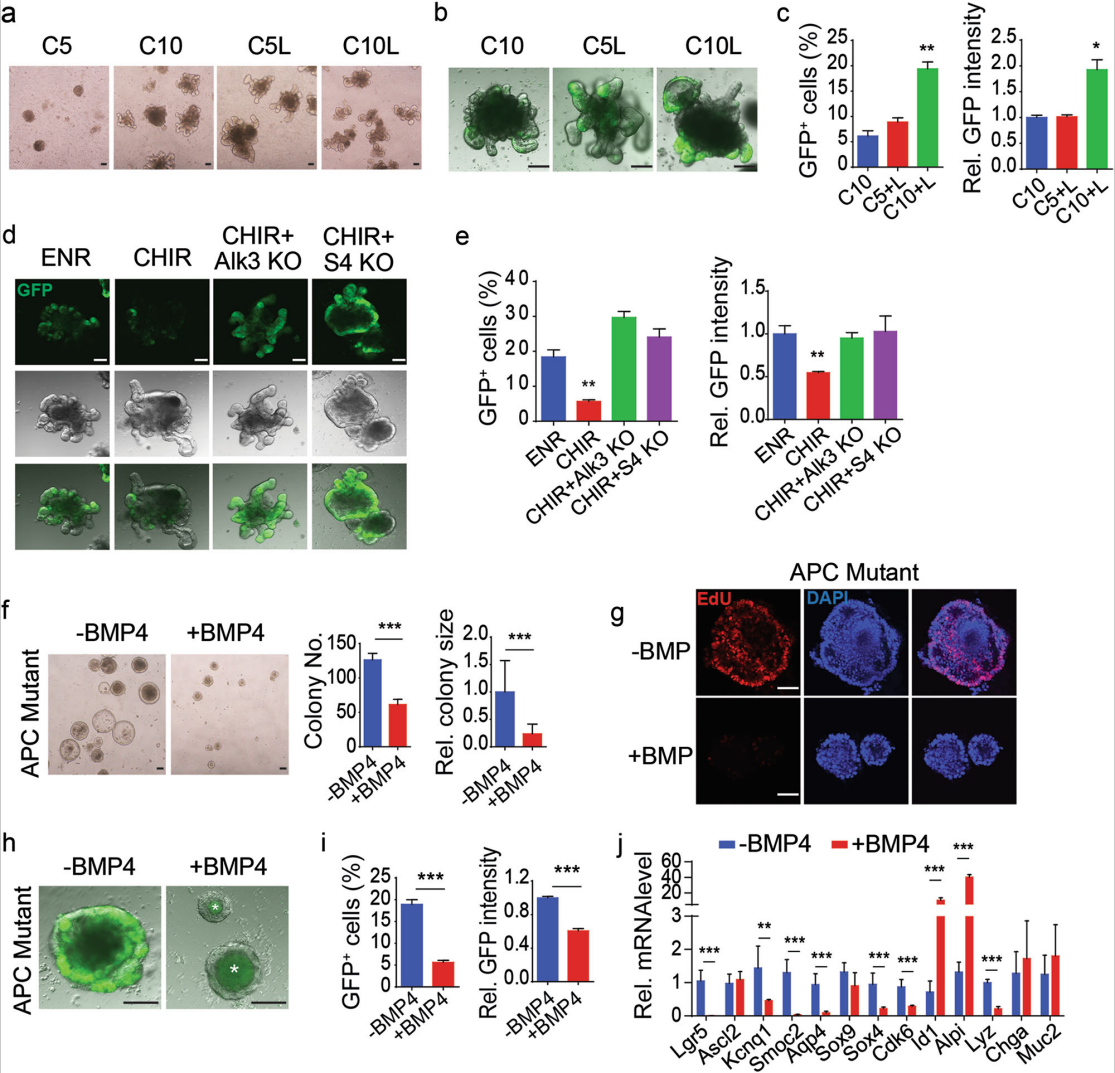
BMP signaling coordinates with Wnt signaling to sustain Lgr5^+^ ISCs. **a** Representative bright-field images of small intestinal organoids cultured for 7 days in 5 μM CHIR (C5), 10 μM CHIR (C10), 5 μM CHIR plus LDN (C5L) and 10 μM CHIR plus LDN (C10L). **b**, **c** Fluorescence images (**b**) and FACS analysis of GFP expression (**c**) of small intestinal organoids cultured with C10, C5 and C10L for 7 days. **d**, **f** Fluorescence images (**d**) and FACS analysis of GFP expression (**e**) of wild-type, *Alk3*^*−/−*^ or *Smad4*^*−/−*^ crypts cultured in the indicated conditions for 7 days. **f** Representative images and quantitation of colony numbers and sizes of *Apc*^*−/−*^ organoids cultured with or without BMP4. **g** EdU staining of proliferating cells in *Apc*^−/−^ organoids cultured with or without BMP4. **h**, **i** Fluorescence images (**h**) and FACS analysis of GFP expression (**i**) of *Apc*^*−/−*^ organoids with or without BMP4. **j** Gene expression change of *Apc*^*−/−*^ organoids after BMP4 treatment. Scale bars, 100 μm. ***P < 0.001, **P < 0.01, and *P < 0.05 analyzed by two-way ANOVA test in **c** and **e** and by Student’s *t*-test in **f**, **i**, and **j**. Error bars, s.d. *n* = 3 mice in **c**, *n* = 4 wells from 2 mice in **e** and **i**, *n* = 3 independent experiments in **f** and **f**, 50 organoids were calculated for their sizes in **f**

The above results indicate that activation of Wnt/β-catenin signaling and inhibition of BMP/Smad signaling are sufficient to maintain the self-renewal of Lgr5^+^ ISCs. We then explored whether the coordination of the two signaling pathways is necessary to sustain Lgr5^+^ ISCs. Low-dose CHIR (5 μM) was unable to support the survival of organoids (Fig. 4a). Although adding LDN or rising CHIR to 10 μM could successfully maintain the organoid survival, the Lgr5^+^ ISCs were well maintained only in the condition with high Wnt/β-catenin signaling activity and abolishment of BMP/Smad signaling (Fig. 4b, c). This is supported by the data that genetic ablation of BMP signaling through disrupting the *Alk3* or *Smad4* gene greatly enhanced the GFP^+^ cells population in CHIR-cultured organoids (Fig. 4d, e). *Apc*-deficient Lgr5^+^ cells could be maintained in the condition absent of Noggin^23^. However, if BMP4 was included in the culture medium, both the colony number and size dramatically decreased (Fig. 4f). In line with this, EdU stained proliferating cells decreased a lot after BMP4 treatment (Fig. 4g). In addition, BMP treatment led to the loss of Lgr5^+^ ISCs in the *Apc* mutant organoids (Fig. 4h, i). Consistently, BMP4 downregulated most Lgr5^+^ ISC signature genes, except *Ascl2* and *Sox9* (Fig. 4j). BMP also altered differentiation marker expression, increasing the enterocyte marker *Alpi* expression while suppressing the Paneth cell marker *Lyz* expression. In summary, our results show that activation of Wnt/β-catenin signaling and inhibition of BMP/Smad signaling are necessary and sufficient to sustain Lgr5^+^ ISC self-renewal in vitro.

Although EGFR signaling is critical for intestinal regeneration and tumor formation^6^, EGF is dispensable as the endogenous EGF activation may be enough to maintain cell proliferation in cultured organoids^13,14^. R-spondin1 can be replaced by the GSK3 inhibitor CHIR, suggesting that the unequal role of Wnt ligands and R-spondin^15^ could be the result of their differential activation ability for Wnt/β-catenin signaling. The successful long-term maintenance of Lgr5^+^ ISCs with the two small molecules CHIR and LDN provides a convenient and cheap culture system for the study and expansion of intestinal stem cells in vitro. This system may also be used for the culture and large quantity expansion of human intestinal stem cells to treat intestinal degeneration diseases.

## Materials and methods

### Mice

*Lgr5-EGFP-IRES-creERT2*^18^ and *Apc*^*fl/fl*^ mice were obtained from Jackson Laboratory. *Alk3*^*fl/fl*^ mice^24^ were kindly provided by Yuji Mishina and *Vil-creERT2* mice^25^ were a gift from Sylvie Robine. *Smad4*^*fl/fl*^ mice^26^ were from Xiao Yang. All mice were back-crossed into the C57BL/6 genetic background for at least 10 generations. The 8- to 16-week-old mice were used for crypts isolation. For gene-ablation experiments, 6- to 8-week-old mice were injected intraperitoneally with 100 μl tamoxifen in sunflower oil at 20 mg/ml for 5 consecutive days. One month later, *Alk3*^*−/−*^*;Lgr5-EGFP-IRES-creERT2;Vil-creERT2* and *Smad4*^*−/−*^*;Lgr5-EGFP-IRES-creERT2;Vil-creERT2* mice were sacrificed for crypts isolation. *Apc*^*−/−*^*;Lgr5-EGFP-IRES-creERT2;Vil-creERT2* mice were used 2–3 months after tamoxifen injection. All animal studies were performed in accordance with the relevant guidelines and under the approval of the Institutional Animal Care and Use Committee of Tsinghua University.

### Isolation of intestinal crypts and organoid culture

Intestinal crypts were isolated and cultured as previously described^22^. Briefly, mouse intestine was cut longitudinally and washed three times with cold PBS. Villi were carefully scraped away and small pieces (5 mm) of intestine were incubated in 2 mM EDTA in PBS for 40 min on ice. These pieces were then vigorously suspended in cold PBS and the mixture was passed through 70 μm cell strainer (BD Biosciences). The crypt fraction was enriched through centrifugation (3 min at 300–400 g). Then the crypts were embedded in growth factor-reduced Matrigel (BD Biosciences, 356231) and seeded on 48-well plate or 24-well plate. After polymerization, crypt culture medium (Advanced DMEM/F12 supplemented with Penicillin/Streptomycin, GlutaMAX-I, N2, B27, and *N*-acetylcysteine (Invitrogen)) containing EGF (50 ng/ml, Invitrogen), Noggin (100 ng/ml, R&D) and R-spondin1 (500 ng/ml, R&D) (ENR) or CHIR-99021 (Selleck, S1263) and LDN-193189 (Selleck, S2618) were added as indicated in the figure legends and refreshed every 2–3 days. For colon culture, 100 ng/ml recombinant Wnt3a protein (R&D, 1324-WN-500/CF) was added in the ENRW group. For passaging, the organoids embedded in Matrigel in each well were directly suspended in 1 ml cold PBS after removal of medium and were pelleted by centrifugation (3 min at 300–400 g). The pelleted organoids were embedded in fresh Matrigel and seeded on plate followed by addition of culture medium as indicated in the figure legends. For single-cell culture, GFP^+^ cells sorted from FACS were embedded in the matrigel and were cultured in the crypt culture medium as indicated in the figure legends. For organoid colony quantification, about 500 crypts were seeded each well and the survived organoids were counted 3 days later. For passaging, the organoids embedded in Matrigel were suspended in 1 ml cold PBS and pelleted by centrifugation (3 min at 300–400 × *g*). The pelleted organoids were embedded in fresh Matrigel and seeded on a plate followed by addition of indicated culture medium. For single-cell culture, 5000 GFP^+^ cells sorted from FACS were cultured with the indicated medium together with 10 μM blebbistatin. In single cell culture, LDN was reduced to 100 nM.

### Immunofluorescence

To monitor GFP expression, the Lgr5-GFP organoids were observed with Olympus FV1200 confocal microscope. For immunofluorescence, cultured organoids were first fixed for 1 h with 4% paraformaldehyde at room temperature. Organoids were washed with PBS in the eppendorf tubes for 3 times. Samples were permeabilized with 2% Triton X-100 for 2 h in the 4 °C and blocked with PBT solution (3%BSA and 1% Triton X-100 in PBS) for 2 h at room temperature. The organoids were then incubated overnight with the primary antibody at 4 °C. The following primary antibodies were used: rabbit anti-lysozyme (Dako, F0372, 1:200), rabbit anti-Muc2 (Santa Cruz, sc-15334, 1:300), goat anti-Chromogranin A (Santa Cruz, sc-1488, 1:300). The fluorescein-labeled secondary antibodies (Life Technologies, 1:300) and 4′, 6-diamidino-2-phenylindole (DAPI) were applied for 1 h at room temperature. 5-Ethynyl-2-deoxyuridine (EdU) staining was performed by following the manufacturer’s instruction (Click-IT; Invitrogen). Images were obtained with an Olympus FV1200 Laser Scanning Microscope.

### Flow cytometry

Fresh intestinal crypts isolated from *Lgr5-EGFP-IRES-creERT2* mice were incubated in TrypLE (Invitrogen) for 20 min at 37 °C to obtain single-cell suspension. The organoids embedded in Matrigel were first suspended in cold PBS after medium discarding, pelleted by centrifugation (3 min at 300–400 × *g*) and then incubated in TrypLE for 20 min at 37 °C to obtain single-cell suspension. The dissociated cells were stained with propidium iodide (PI) and passed through 40 μm cell strainer (BD), and then single GFP-high cells were analyzed or sorted by flow cytometry (MoFlo XDP, Beckman). PI-negative cells were gated as GFP-positive and GFP-negative populations and analyzed with Summit 5.3 or Flowjo software.

### Chromosome analysis

For karyotyping, organoids were pre-treated with colchicine for 6 h before harvested for single cell dissociation using TrypLE. After centrifugation, cell pellets were resuspended with 75 mM KCl and incubated for 15 min at room temperature. Cells were centrifuged, fixed in methanol:acetic acid (3:1) on ice and dropped onto glass slides. Air-dried cells were stained with Giemsa staining, and metaphase spreads were imaged and analyzed. A total of ten metaphases were examined and all had the normal karyotype.

### RNA extraction and qRT–PCR

Total RNA was extracted with RNeasy Mini Kit (Qiagen) according to the manufacturer’s instruction. cDNA was prepared using Revertra Ace (Toyobo). qRT–PCR was performed with TransStart Green qPCR SuperMix (Transgen Biotech) in triplicates on a LightCycler 480 (Roche) with Gapdh as the reference gene. Data were analyzed according to the ΔCT method.

### RNA-seq

Organoids derived from *Lgr5-EGFP-IRES-creERT2* mice were cultured in the medium containing ENR or CL for 2 months. The medium was refreshed every 2–3 days, and the organoids were passaged normally. The organoids were dissociated into single cell suspension and single GFP^high^ cells were then sorted by flow cytometry. RNA was purified from 200,000 single GFP^high^ cells using the RNeasy Mini Kit (Qiagen) and converted into cDNA libraries using the Ovation® RNA-Seq System V2 kit (NuGEN). High-throughput sequencing was performed using the Illumina HiSeq X Ten. The RNA-seq was carried out with two biological replicates. The RNA-seq data were uniquely mapped to mm9 genome by Hisat2 (version 2.0.5). The gene expression was calculated by StringTie (version 1.3.1c) using the refFlat database from the UCSC genome browser.

### Immunoblotting

Protein lysates were prepared from organoids, and immunoblotting was performed as previously described^22^. The following primary antibodies were used: mouse anti-β-catenin (Sata Cruz, sc-7963, 1:1000), rabbit anti-Smad1 (CST, 6944, 1:2000), rabbit anti-p-Smad1/5/8 (CST, 9511, 1:2000), rabbit anti-p-AKT^473^ (CST, 4060, 1:2000), rabbit anti-AKT (CST, 9272, 1:2000), mouse anti-YAP (Sata Cruz, sc-101199, 1:1000), rabbit anti-p-p38 (CST, 9215L, 1:2000), mouse anti-P38 (Sata Cruz, sc-7972, 1:1000), and mouse anti-tubulin (Proteintech, 66031–1-Ig, 1:20,000).

### Reporter assay

Cells were transfected with various plasmids as indicated, and at 12 h post-transfection, the cells were treated with small molecules or growth factors. Twenty-four hours later, the cells were harvested for luciferase determination with aluminometer (Berthold Technologies). Reporter activity was normalized to the co-transfected Renilla. For the Topflash reporter, the activity was normalized to Fopflash. Experiments were independently repeated at least three times.

### Statistics

Data shown in column graphs represent the mean ± s.d. When normality could be assumed, Student’s *t*-test or two-way ANOVA analysis was used to compare difference between two groups as indicated in the figure legends. *P < 0.05, **P < 0.01, ***P < 0.001. Statistical analysis was performed with GraphPad Prism6 software. The quantification of colony size was performed with ImageJ. Each experiment was independently repeated at least three times.

## Electronic supplementary material

Supplemental Information

## Electronic supplementary material

**Supplementary Information** accompanies the paper at (10.1038/s41421-018-0051-0).
