# Supplementary material for: A growth factor-free culture system underscores the coordination between Wnt and BMP signaling in Lgr5+ intestinal stem cell maintenance
Source: Cell Discov. 2018 Sep 4;4:49. doi: 10.1038/s41421-018-0051-0 (PMC6120946; doi:10.1038/s41421-018-0051-0)
Supplement: Supplementary file 1 — Supplemental Information [file 41421_2018_51_MOESM1_ESM.pdf]

Supplementary Information

# A growth factor-free culture system underscores the coordination between Wnt and BMP signaling in Lgr5$^+$ intestinal stem cell maintenance

Yehua Li[1*], Yuan Liu[1*], Bofeng Liu[2], Jilian Wang[3], Siting Wei[1], Zhen Qi[1], Shan Wang[1], Wei Fu[3], Ye-Guang Chen[1#]

Supplementary Figure 1-4

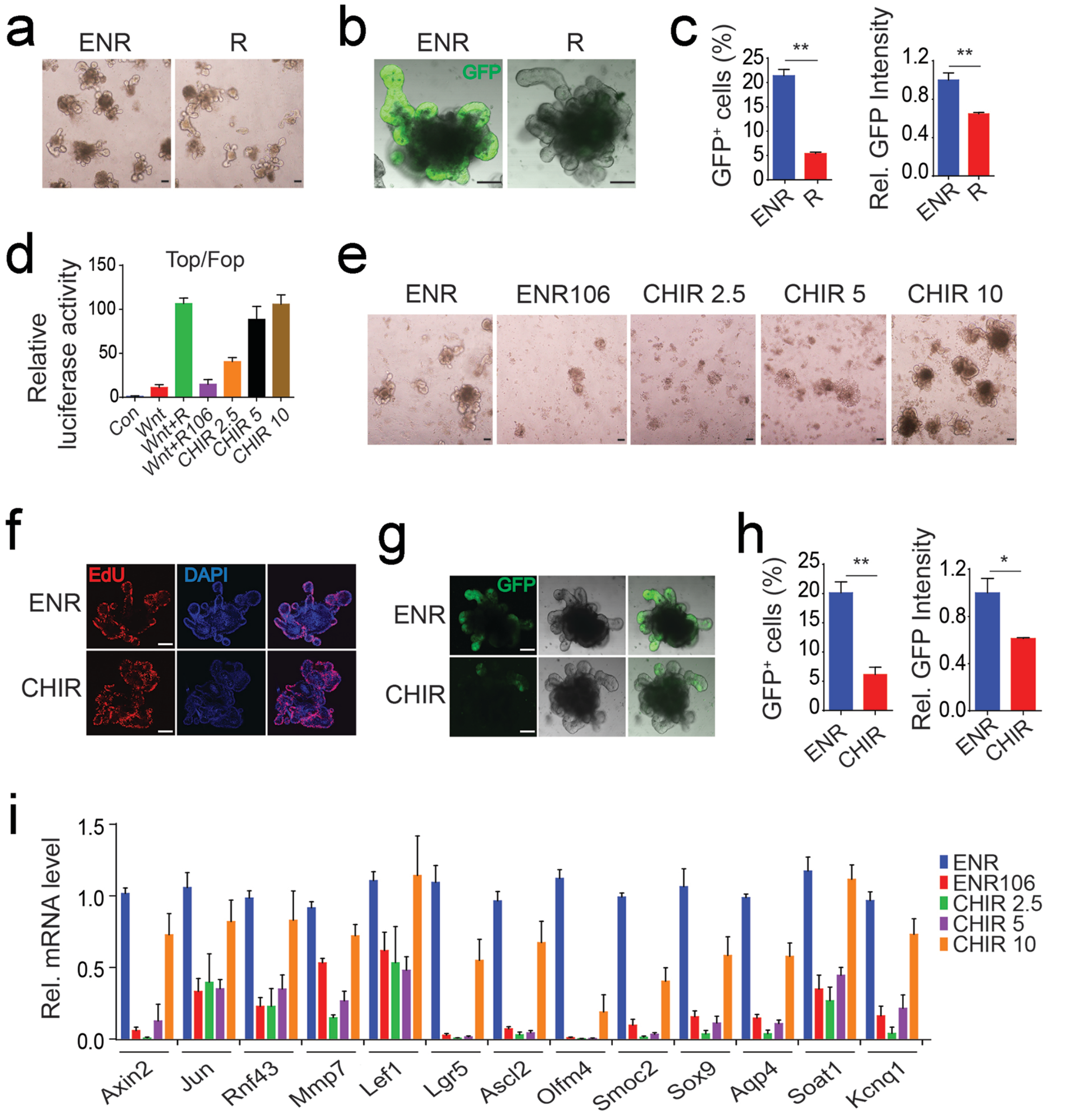


**Supplementary Figure 1. CHIR can substitute R-spondin1 in small intestinal organoid culture. a** Representative bright-field images of small intestinal organoids cultured with EGF, Noggin and R-spondin1 (ENR) and only R-spondin1 (R) for 7 days. **b,c** Fluorescence images (**b**) and FACS analysis of GFP expression (**c**) of small intestinal organoids cultured with ENR and R for 7 days. **d** HEK293T cells were transfected with Topflash-Luc or Fopflash-Luc and then treated with Wnt3a, Wnt3a plus R-spondin1 (Wnt+R), Wnt3a plus R-spondin1(F106A) mutant (Wnt+R106), 2.5μM CHIR (CHIR 2.5), 5μM CHIR (CHIR 5) and 10μM CHIR (CHIR 10) for 24

hours before harvested for luciferase activity determination. **e** Representative bright-field images of small intestinal organoids cultured with ENR, CHIR 2.5, CHIR 5, CHIR 10 and EGF, Noggin plus R-spondin1(F106A) for 7 days. **f** EdU staining of proliferation cells in small intestinal organoids cultured with ENR and CHIR for 7 days. **g,h** Fluorescence images (**g**) and FACS analysis of GFP expression (**h**) of small intestinal organoids cultured with ENR and 10μM CHIR for 7 days in. **i** Gene expression of small intestinal organoids cultured with the indicated conditions for 7 days. Scale bars, 100 μm. **P < 0.01 and *P < 0.05 analyzed by Two-way ANOVA test in (**c**) and (**h**). Error bars, s.d. n = 3 mice in (**c**) and (**h**), and n=3 independent experiments in (**d**) and (**f**).

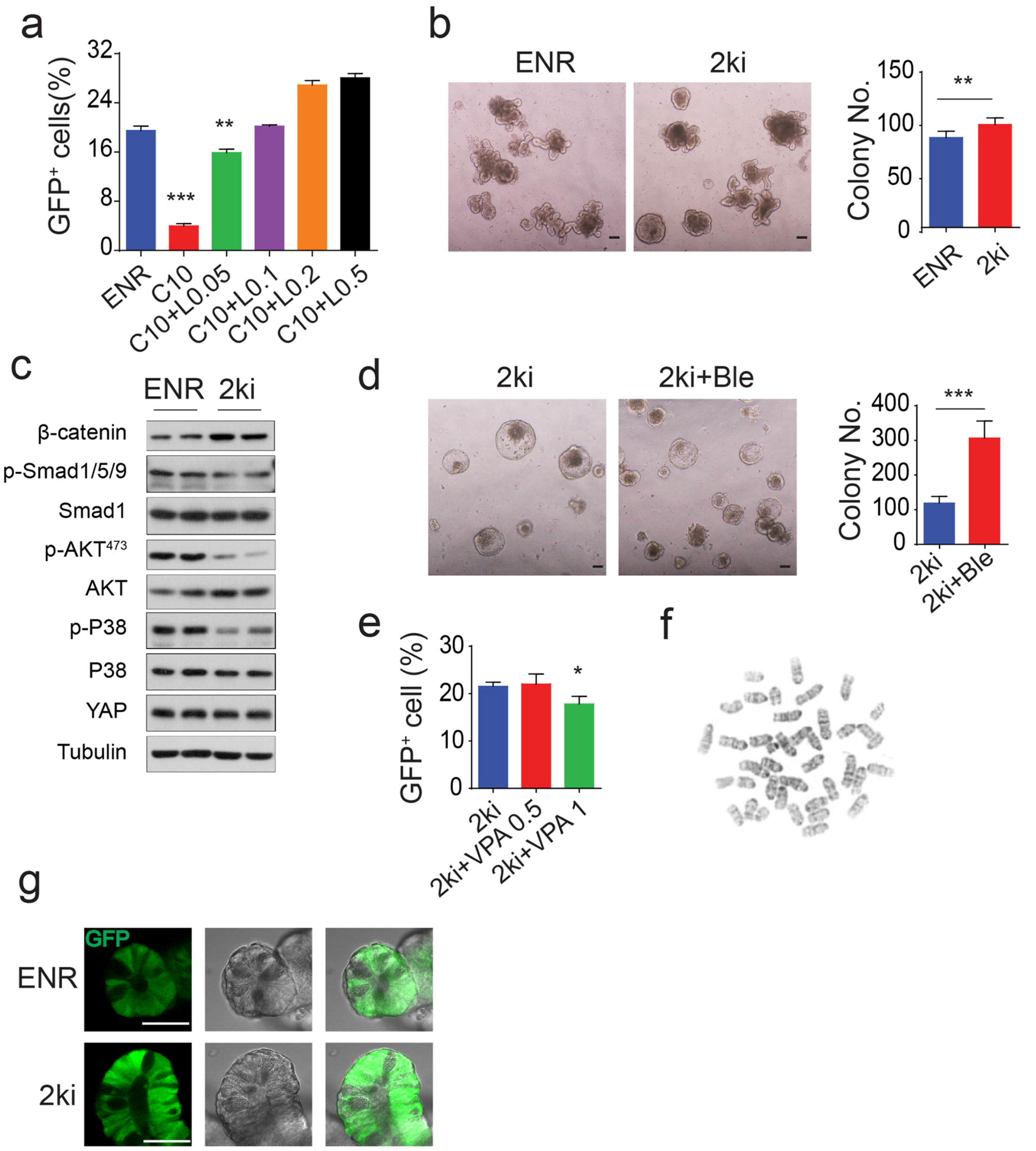


**Supplementary Figure 2. Comparison of organoids cultured with ENR or 2ki. a** FACS analysis of GFP expression of small intestinal organoids cultured for 7 days in ENR 10μM CHIR or with different concentration of LND. **b** Representative images and quantitation of colony numbers of small intestinal organoids cultured with ENR and 2ki for 7 days. **c** Immunoblotting of protein levels in ENR- or 2ki-cultured organoids. **d** Representative bright-field images and quantitation of colony numbers of organoids cultured in 2ki or 2ki plus 10μM blebbistatin for 4 days. **e** GFP

expression of small intestinal organoids cultured with 2ki or 2ki plus 0.5 mM or 1 mM valproic acid for 7 days. **f** Karyotyping of a cell cultured with 2ki for 2 months. **g** GFP fluorescence images of budding from small intestinal organoids cultured with ENR and 2ki for 2 months. Scale bars, 100 μm in **b** and **d**, 50μm in **g**. ***P < 0.001 and **P < 0.01 analyzed by Two-way ANOVA test in (**b**) and (**d**) and by Student's t-test in (**a**) and (**e**). Error bars, s.d. n = 9 wells from 3 mice in (**b**) and (**d**), and n=3 wells in (**a**) and (**e**).

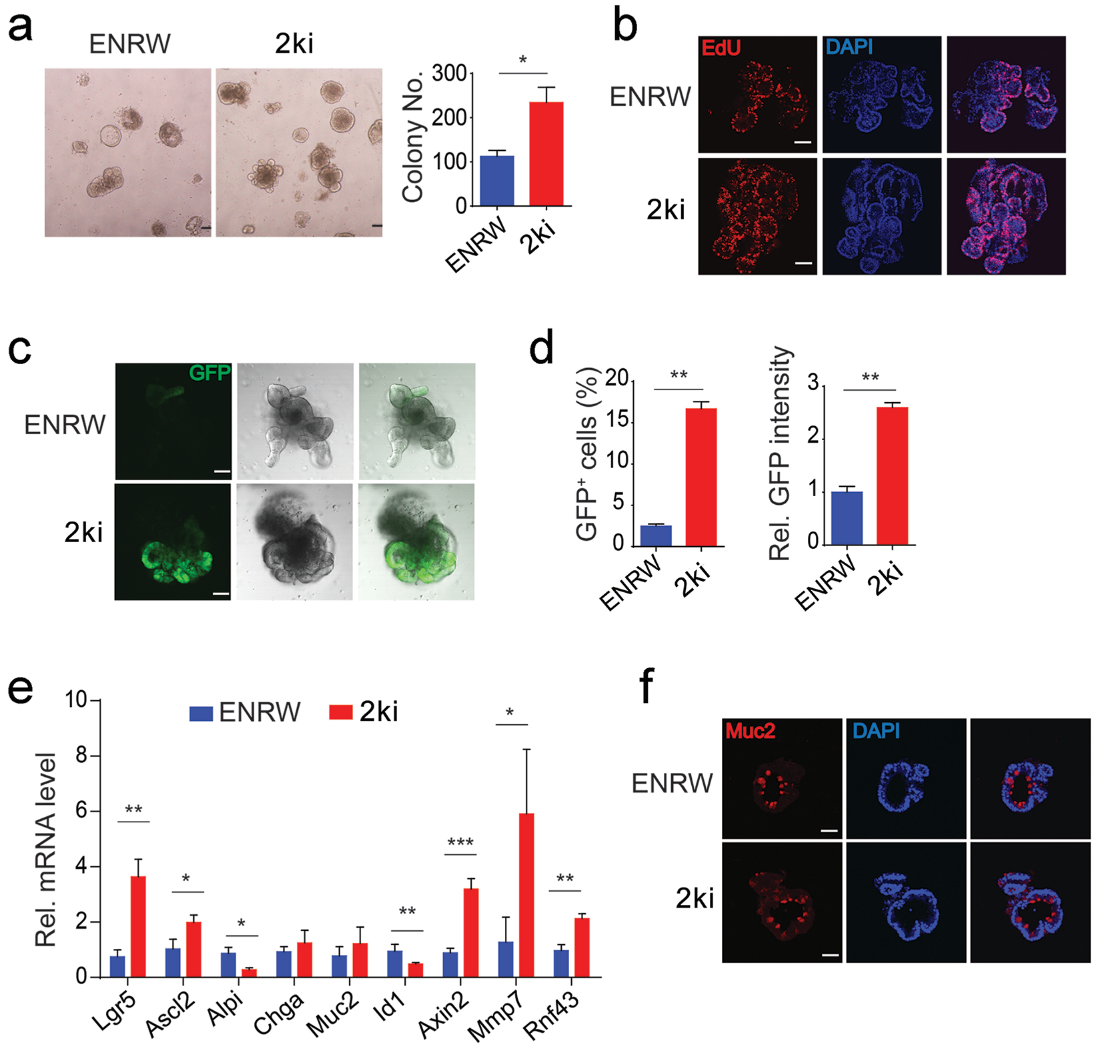


**Supplementary Figure 3. CHIR and LDN together sustain the self-renewal of ISCs from colon crypts. a** Representative images and quantitation of colony numbers of colon organoids cultured with EGF, Noggin, R-spondin1 and Wnt3a (ENRW) or CHIR and LDN (2ki). **b** EdU staining of proliferation cells in colon organoids cultured with ENRW and 2ki for 7 days. **c,d** Fluorescence images (**c**) and FACS analysis of GFP expression (**d**) of colon organoids cultured for 7 days. **e** Gene expression of colon organoids cultured for 7 days. **f** The goblet cell marker Mucin 2 staining in colon organoids cultured for 7 days. Scale bars, 100 μm. ***P < 0.001, **P<0.01 and *P < 0.05 analyzed by Two-way ANOVA test in (**b**) and (**d**) and by

Student's t-test in (**e**). Error bars, s.d. n = 3 mice in (**b**) and (**d**), and n=3 independent experiments in (**e**).

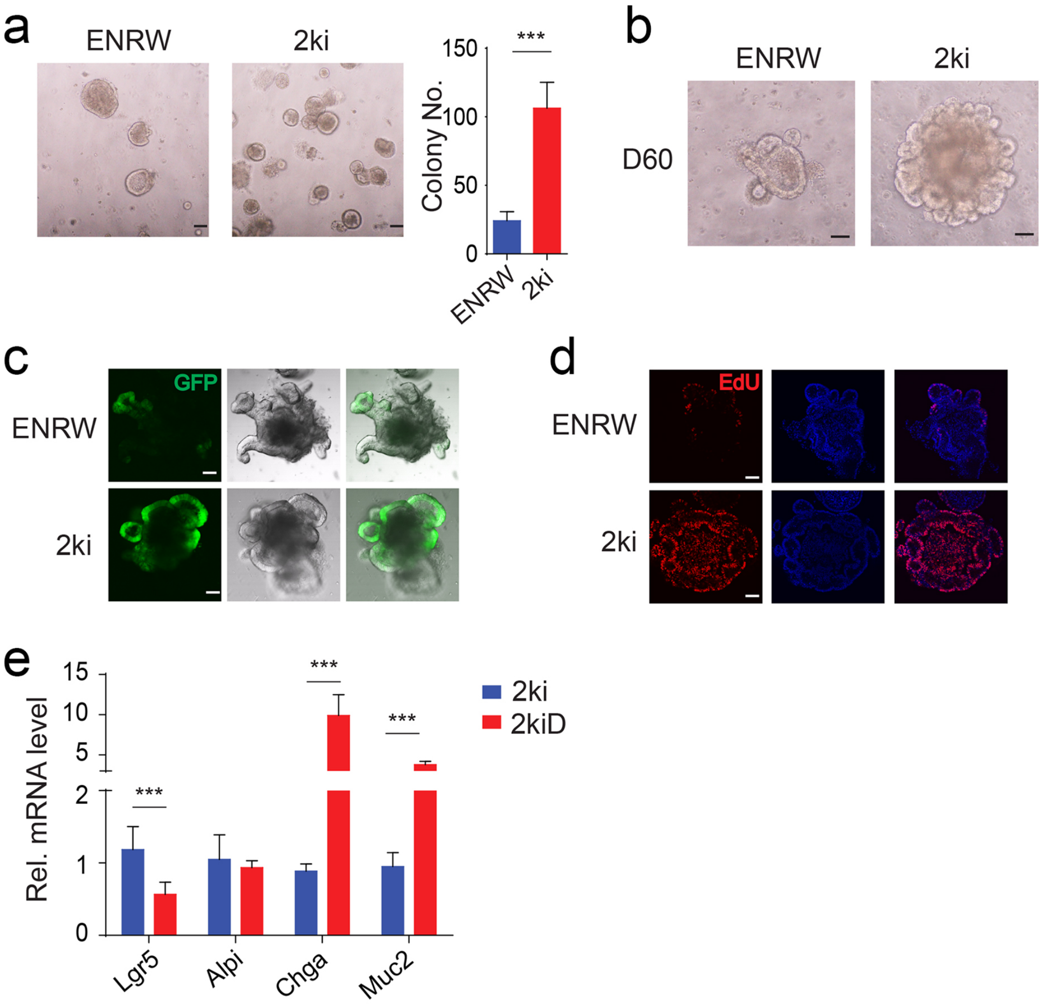


**Supplementary Figure 4. Long-term maintenance of colonic Lgr5$^+$ ISCs in 2ki condition. a** Representative images and quantitation of colony numbers of 5,000 FACS-isolated colon Lgr5-GFP+ cells cultured with ENR plus Wnt3a (ENRW) or CHIR and LDN (2ki) for 7 days. **b-d** Representative bright-field (**b**), GFP fluorescence images (**c**) and EdU staining of proliferating cells (**d**) of colon organoids cultured with ENR and 2ki for 2 months. **e** Gene expression of stem cell and differentiated cell marker genes in colon organoids cultured with 2ki or 2ki+DAPT for 4 days. Scale bars, 100 μm. ***P<0.001 analyzed by Two-way ANOVA test in **a** (n=9 wells from 3 mice) and by Student's t-test in **e** (n = 3 independent experiments). Error bars, s.d..
